# Validation of a simple diagnostic relationship for downslope flows

**DOI:** 10.1002/asl.965

**Published:** 2020-03-02

**Authors:** Joan Cuxart, Daniel Martínez‐Villagrasa, Ivana Stiperski

**Affiliations:** ^1^ Department of Physics University of the Balearic Islands Palma Spain; ^2^ Department of Atmospheric and Cryospheric Sciences University of Innsbruck Innsbruck Austria

**Keywords:** diagnostic formula, downslope flow, katabatic depth, MATERHORN campaign, METCRAX II campaign

## Abstract

A simple conceptual view of downslope flows allows a derivation of a diagnostic relationship between the maximum depth of the flow, its speed, the slope angle and the cooling flux. This relationship is obtained considering that the turbulence cooling causing the air to flow downslope is essentially compensated by the warming by compression as the flow reaches higher pressure levels. The obtained relationship is consistent with the bulk heat budget for the along‐slope flows, and its agreement with existing prognostic layer‐averaged models is checked. Finally, the depth of the flow obtained from the diagnostic relationship is compared against the observational estimations of katabatic flows from two experimental campaigns.

## Graphical abstract

A simple formula for downslope flows is presented and compared to recently obtained experimental data. It relates the wind speed, the slope angle, the surface cooling flux, the depth of the flow, and the background thermal stratification. It is consistent with previous findings based on the bulk heat budget equation for the along‐slope wind. The figure shows the variation of the depth of the downslope flow (*D*_max_) allowing only one variable to change. The values for the variables when fixed are *v* = 2 m·s^−1^, *H* = −0.01 K·m·s^−1^, *∂*_*z*_ *θ* = 0.0033 K·m^−1^, and *α* = 15°.

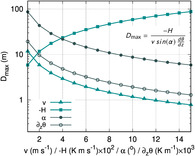

## 1 INTRODUCTION

Slope flows have been analyzed for more than a century and are well described by a number of theories, many of them summarized in the work by Zardi and Whiteman (2013) and their interaction with the general features of the mountainous Atmospheric Boundary Layer has been recently reviewed by Serafin *et al*. (2018). The basic characteristics of these flows are well understood, considering that air flowing downslope warms by compression and cools in contact with the surface. Downslope (or katabatic) flows have been studied traditionally using the momentum equation in natural coordinates along and normal to the slope (Mahrt, 1982). This method implies the closure of the surface energy budget and the use of a gradient approach for the turbulence fluxes, imposing a functional form for the eddy diffusivity as in Grisogono and Oerlemans (2001).

In this study, a local diagnostic relationship for downslope flows is obtained relating some basic parameters of the problem, namely surface cooling, slope angle, downslope speed, background stability and depth of the flow. Simplifications are made, similar to those assumed in previous approaches, such as Fleagle's layer‐averaged concept (Fleagle, 1950), further developed by Doran and Horst (1981) or McNider (1982), or the simpler approach of Kondo and Sato (1988). Entrainment across the top of the downslope flow is not considered here. It can be a relevant mechanism (Manins and Sawford, 1979), although it is presumably small when a well‐defined thermal inversion tops the flow (Martínez and Cuxart, 2009).

There are still some discussions on how to define the depth of a downslope flow. Some possibilities are the height of the maximum speed, the whole depth of air flowing downward, or the layer with presence of turbulence. In the simple approach here, the depth is taken as the size of the largest turbulent eddies and comparison to other approaches will favor definitions compatible with this one, in particular using the databases of the campaigns MATERHORN (Fernando *et al*., 2015) and METCRAX II (Lehner *et al*., 2016). Furthermore, comparison with these data will also allow to explore the adequacy of the hypotheses made by comparing a value of the depth estimated from the observations.

In this document, the simple physical principles leading to the proposed expression will be described in Section 2, the consistency with already existing formulations in the literature will be sought in Section 3 and a comparison test with observations will be made in Section 4, before the final concluding section.

## 2 DERIVATION OF A DIAGNOSTIC RELATIONSHIP

Consider a parcel of air, with temperature *T*_p_, over a slope with angle *α* experiencing radiative cooling transferred upwards through the turbulent heat flux (*H* < 0, in K·m·s^−1^) over a depth *D*, which represents in this scheme the size of the largest turbulent eddies performing the transport. As the parcel becomes negatively buoyant compared to the environment air further away from the surface, with temperature *T*_e_, it begins to flow downhill with a change of elevation Δ*z* < 0, with *z* taken positive upwards.

The cooling along a distance *d* over the slope, with speed *v* during a time *t*, can be estimated as

(1) $$\Delta T_{c}=\frac{H}{D}t=\frac{H}{D}\frac{d}{v}=-\frac{H}{D}\frac{\Delta z}{v\sin\left(\alpha \right)}$$

On the other hand, as the particle reaches lower elevations, the warming by adiabatic compression is

(2) $$\Delta T_{\mathrm{ad}}=-\frac{g}{\mathrm{Cp}}\Delta z$$

At this point, the parcel will continue flowing downhill if it is colder than the environment

(3) $$T_{\mathrm{pi}}+\Delta T_{c}+\Delta T_{\mathrm{ad}}<T_{\mathrm{ef}}$$

where *T*_pi_ is the initial temperature of the particle and *T*_ef_ is the environment temperature at destination.

Assuming that *T*_pi_ is approximately equal to *T*_ei_, which is the temperature of the environment at the initial point, it leads to

(4) $$\Delta T_{c}+\Delta T_{\mathrm{ad}}<T_{\mathrm{ef}}-T_{\mathrm{ei}}\equiv \Delta T_{e}$$

Dividing by Δ*z*, switching to differentials and introducing *θ* through

(5) $$\frac{T}{\theta }\frac{\mathrm{d\theta}}{\mathrm{dz}}=\frac{\mathrm{dT}}{\mathrm{dz}}+\frac{g}{C_{p}}$$

the following inequality is obtained after assuming a shallow flow approach ($\frac{T}{\theta }\approx 1$)

(6) $$-\frac{H}{D}\frac{1}{v\sin\left(\alpha \right)}>\frac{\mathrm{d\theta}}{\mathrm{dz}}$$

If the strong hypothesis is made that *D*, besides being the depth of the layer which is experiencing surface cooling, is also the depth of the downslope flow, the above inequality leads to an expression for an upper value for *D*, namely

(7) $$D_{\max}=\frac{-H}{v\sin\left(\alpha \right)\frac{\mathrm{d\theta}}{\mathrm{dz}}}$$

Since *D*_max_ is conceptually introduced as the height of the largest turbulent eddies, it is an approximation to the height of the turbulent layer associated with the existence of a downslope flow. This relationship, simply obtained by comparing two main mechanisms, surface cooling transported upwards by turbulence and adiabatic warming by compression, is equivalent for the downslope flows to the one derived by Vergeiner and Dreiseitl (1987) using the along‐slope mass flux and the heat input at the surface. Schmidli (2013) analyzed that expression for the daytime upslope flows with data from Large‐Eddy Simulations, using the mass flux as the quantity to determine.

**Figure 1:**
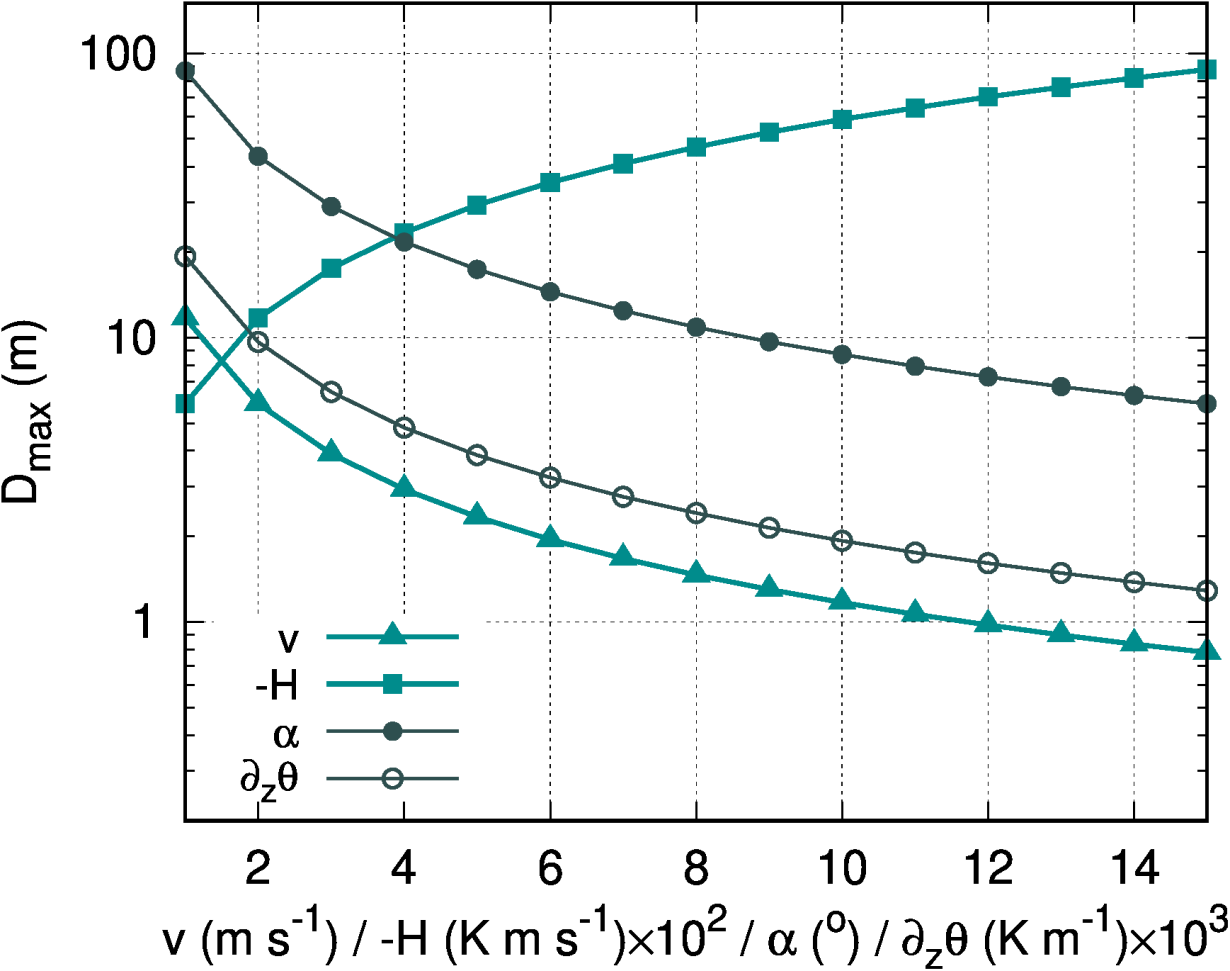
Variation of the depth of the downslope flow (*D*_max_) allowing only one variable to change. The values for the variables when fixed are *v* = 2 m·s^−1^, *H* = −0.01 K·m·s^−1^, *∂*_*z*_ *θ* = 0.0033 K·m^−1^ and *α* = 15^°^

Figure 1 illustrates the behavior of *D*_max_ when only one parameter varies and the others stay fixed (taking a slope of 15°, a speed of 2 m·s^−1^, a heat flux of −0.01 K·m·s^−1^ and stratification $\frac{\mathrm{d\theta}}{\mathrm{dz}}$ = 0.0033 K·m^−1^). For downslope flows of low speeds (in the range 1–3 m·s^−1^) over this relatively steep slope (15^°^) the depth decreases with speed from values just above 10 m to about 5 m. Larger speeds generate very shallow flows (between 0.5 and 2 m), indicating that at that point of the slope the contact time with the surface is too short to allow for a deep cooling layer. Similarly, small inclinations (below 3°) have depths of some tens of meters under low speed conditions, showing that the cooling can be transferred upwards efficiently at the point of interest. As the inclination increases, the contact time with the surface decreases as does *D*_max_.

On the other hand, the larger the cooling rate the deeper the downslope flow, if all other factors are kept constant. For the usual ranges in the nocturnal boundary layer (up to −0.03 K·m·s^−1^) the depth is in the range 5–15 m. If the fluxes were much more intense, the depth could increase significantly. Contrarily, increasing the thermal stability of the atmosphere at the layers close to the surface limits the vertical extent of the downslope flow, due to the associated very weak turbulence, and can generate very shallow flows when the stability becomes strong.

The proposed relationship is a diagnostic formula only valid for slope‐generated flows. In principle it should not be used when other forcings are in play, like when mesoscale pressure gradients are important drivers, such as in mesoscale basin flows (Cuxart, 2008), mountain‐plain flows (Jiménez and Cuxart, 2014), or valley flows under a valley‐plain pressure gradient (Jiménez *et al*., 2019).

## 3 CONSISTENCY WITH PREVIOUS LAYER‐AVERAGED MODELS

The diagnostic Equation (7) can also be derived from the temperature tendency equation of a descending particle

(8) $$\frac{\mathrm{d\theta}}{\mathrm{dt}}=\frac{\partial \theta }{\partial t}+v\frac{\partial \theta }{\partial x^{\prime }}+w\frac{\partial \theta }{\partial z^{\prime }}=-\frac{1}{\rho_{0}C_{p}}\frac{\partial Q^{*}}{\partial z^{\prime }}-\frac{\partial H}{\partial z^{\prime }}$$

where the term on the right hand side represents the heating/cooling rate due to the vertical flux divergences of net radiation (Q*) and kinematic heat flux (*H*), respectively. Primes on *x* and *z* indicate that the coordinate axis is rotated parallel and normal to the slope surface.

To reach the diagnostic formulation, we can consider a layer‐averaged model and write Expression (8) in terms of the bulk temperature deficit △ (i.e., Martínez and Cuxart, 2009), which represents the integrated difference between the extrapolated potential temperature of the background air *θ*_0_ and the potential temperature *θ* within the cooled layer above the surface. In addition, if we: (a) assume that the temperature of the background air is stationary $\left(\frac{\partial \theta_{0}}{\partial t}=0\right)$, (b) consider the motion normal to the slope negligible (*w* ≈ 0), (c) write *∂θ*_0_/*∂x* ^*′*^ = −*∂θ*_0_/*∂z*·sin(*α*) and neglect variations of △ along the slope (*∂*△/*∂x* ^′^ ≈ 0), (d) consider that the radiative cooling has a minor effect on the katabatic layer, and (e) describe the divergence of the kinematic heat flux in this layer as in previous section (−*∂H*/*∂z* ^′^ ≈ *H*/*D*), it leads to

(9) $$\frac{\partial △}{\partial t}=-\frac{H}{D}-v\sin\left(\alpha \right)\frac{\partial \theta_{0}}{\partial z}$$

In this light, there will be a downslope flow if the temperature deficit increases with time or remains constant (*∂*△/*∂t* ≥ 0). If we ignore the tendency term then we recover the diagnostic Relation (7) and, to have a katabatic flow, the diabatic cooling due to divergence of the turbulent heat flux ($-\frac{H}{D}$) must be larger than the warming effect due to the upstream air advected by the katabatic flow itself ($v\sin\left(\alpha \right)\frac{\partial \theta_{0}}{\partial z}$). Note that both of these terms within the parentheses are positive since *H* < 0 and *∂*_*z*_ *θ*_0_ > 0 under stably stratified conditions.

The identification of both cooling/warming rates as the main driving mechanisms for a parcel of air over a sloping surface has been described previously in the literature of katabatic flows. Mahrt (1982) mentions Prandtl (1942) and Defant (1949) as early works whose solutions employ (among other things) a simple thermodynamic relationship where the diffusion of heat is balanced by temperature advection associated with the basic state stratification.

Layer‐averaged models may also combine the thermodynamic Equation (9) with a simplified form of the momentum equation, the latter typically written as (Doran and Horst, 1981)

(10) $$\frac{\partial v}{\partial t}=g\frac{△}{\theta_{0}}\sin\left(\alpha \right)+F_{c}$$

where the first and second terms on the right hand side represent the buoyancy and frictional forces, respectively. McNider (1982), considering the frictional drag as a linear function of *v*, was able to derive an analytical solution for the system of two equations, where the velocity of the katabatic layer oscillates, with a frequency and an intensity that depend on the background stratification.

Doran and Horst (1981) solved the two‐equation system numerically, parameterizing the frictional drag as a function of the wind speed, *D* and a coefficient of entrainment of ambient air into the cooled layer, and describing the diabatic cooling term as −*H*/*D*, as proposed for the diagnostic Expression (7). They obtained oscillatory solutions damped through time for both fields of *v* and potential temperature deficit △, with an oscillation period centred around 1.5–2.0 hr (see their Figure 2). Taking the following values for the input parameters: *D* = 100 m; *α* = 7.2*°*; *θ*_0_ = 290 K; *∂θ*_0_/*∂z* = 0.0016 K·m^−1^; *H* = −0.013 K·m·s^−1^; the resulting wind speed oscillates around a value of *v* ≈ 0.7 m·s^−1^ with a rapid drop of the amplitude with time. Introducing the same input parameters in Expression (7), considering *D* = *D*_max_, the wind speed obtained is *v* = 0.65 m·s^−1^, a very similar value. Therefore, when the transient oscillatory behavior of the wind and temperature associated with the tendency term vanishes, the system converges into a scaling relationship between the katabatic wind speed, slope angle, ambient stratification and diabatic cooling that can be described by Expression (7).

**Figure 2:**
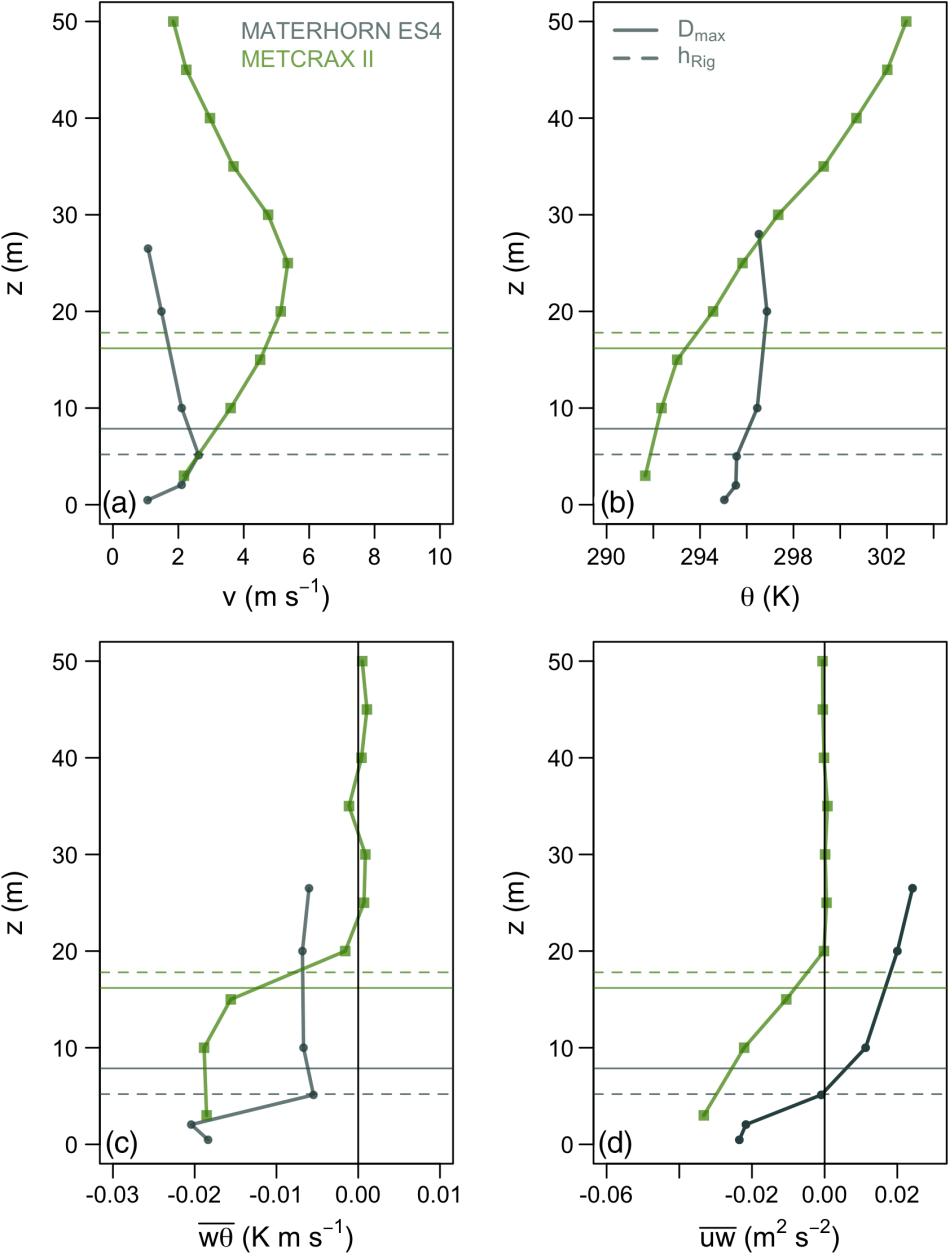
Profiles of (A) wind speed, (B) potential temperature, (C) heat, and (D) momentum fluxes for towers ES4 (MATERHORN) and NEAR (METCRAX II) during representative pure katabatic events. Horizontal lines indicate the diagnostic length scale *D*_max_ (solid) and SBL height $h_{{\mathrm{Ri}}_{g}}$ (dashed) calculated with the experimental data from the two datasets, accordingly identified with different colors

## 4 TEST OF THE DIAGNOSTIC FORMULA AGAINST OBSERVATIONS

The measurements from several meteorological towers are used to explore the validation of the diagnostic formula derived in Section 2. These observations are part of two field campaigns focusing on the study of the flow processes over complex terrain: the Mountain Terrain Atmospheric Modeling and Observation (MATERHORN, Fernando *et al*., 2015), and the second Meteor Crater Experiment (METCRAX II, Lehner *et al*., 2016). Both datasets include observations of katabatic flows of different depth that develop over slopes with slope angles ranging from 1*°* to 6*°*.

During the MATERHORN experiment, four towers were deployed along the eastern slope of the Granite Mountain, each with several levels of instrumentation including sonic anemometers between 0.5 and 20 or 28 m above the surface, depending on the tower (see Figures 1 and 2 in Grachev *et al*., 2016). Here we use mast ES5, located highest on the mountain slope with a local slope angle of 6.4*°*, ES4 that is 600 m away downslope with local slope angle of 4*°*, and ES3 at 700 m further on the same line with slope angle 3*°*. Grachev *et al*. (2016) analyzed data gathered during the Fall experiment in 2012, selecting dry fair weather periods prone to the development of katabatic flows. Observations usually displayed a wind maximum between 2 and 3 m·s^−1^ at heights of about 5 m above the surface, with significant turbulent fluxes below and above the wind maximum.

The NEAR tower was set up during METCRAX II to monitor the mesoscale katabatic flow that regularly forms over the slightly tilted plain surrounding the crater, with a slope of 1*°* which is approximately homogeneous over an upstream distance of 30 km. The drainage wind has a maximum wind speed between 4 and 6 m·s^−1^ within the lowest 50 m above ground (Savage *et al*., 2008). The 50‐m high tower, located about 1.6 km upstream of the crater, was instrumented with 3D sonic anemometers and hygrothermometers at 5‐m intervals to provide vertical profiles of wind, temperature, humidity and turbulent fluxes of momentum and sensible heat.

To assess the proposed diagnostic expression for *D*_max_, it is necessary to compare it with observational data for a well developed pure katabatic flow. Therefore, for the MATERHORN dataset, we analyze the time periods that coincide with those studied by Grachev *et al*. (2016), focusing on the three towers mentioned above, which are the least affected by the circulations developing on the plain. The selected time periods correspond to three consecutive half‐hourly averages between 0200 and 0330 mountain standard time (MST) for three different nights (28/09, 30/09, and 02/10).

For the METCRAX II dataset, the analysis focuses on the data provided by the NEAR tower during the nights (from 1930 to 0400 MST) of the six out of seven Intensive Observing Periods (IOPs) selected during the October 2013 month‐long field campaign that were characterized by undisturbed katabatic flows. The same periods were studied in Stiperski *et al*. (2020) but here data have been additionally filtered by using some thresholds on ambient stability, heat flux and wind speed to focus on pure katabatic flow cases only (see more details below).

To calculate the diagnostic expression *D*_max_ with the selected experimental datasets, the following decisions are taken after exploration of different possibilities: (a) the turbulent heat flux (*H*) is the one closest to the surface; (b) the background potential temperature gradient (*∂θ*_0_/*∂z*) is computed from potential temperature profiles sufficiently high above the jet maximum (10–20 m for MATERHORN and 100–200 m for METCRAX II); (c) the wind speed (*v*) is the maximum value of the observed jet.

Two different profiles representative of the corresponding katabatic events observed in ES4 (MATERHORN) and NEAR (METCRAX II) towers on October 2, 2012 and October 6, 2013, respectively, are shown in Figure 2, together with the corresponding *D*_max_ (solid horizontal lines) obtained from the observations. A shallow downslope flow with a jet maximum of 2 m·s^−1^ at a height near 5 m above ground level (agl) develops at ES4, with speeds becoming significantly weaker at about 20–25 m agl. The temperature profile from the standard slow thermometers shows a change of slope across the jet maximum. The heat fluxes are stronger below than above the jet maximum (as in Conangla and Cuxart, 2006) and the momentum fluxes change sign at the same level. Therefore, it seems that the height of the maximum speed indicates a very well‐defined scale, and the height obtained with *D*_max_ is slightly above this level.

A deeper katabatic wind develops for METCRAX II, with a maximum wind speed above 5 m·s^−1^ at around 25 m agl. The ground‐based temperature inversion is also deeper, covering the entire depth of the drainage flow with increasing stability at several meters away from the surface, where the heat and momentum fluxes become very small. For this case, *D*_max_ is below the maximum wind speed, but it coincides with the level where the stability increases, the turbulent heat flux decreases and momentum flux becomes negligible. Figure 2 shows that the height of the jet maximum is a relevant length scale (cf. Forrer and Rotach, 1997) for the shallow katabatic flows of MATERHORN but, for the deeper drainage winds observed in METCRAX II, the turbulence regime seems to change well below this level.

*D*_max_, defined as the size of the largest turbulent eddies, is specially suitable to be compared to an independent estimation of the depth of the turbulent layer, such as the one given by Stiperski *et al*. (2020) based on the gradient Richardson number (Ri_g_). This parameter requires the mean air temperature and wind speed profiles, which are determined after fitting analytical functions into the multiple observations from the towers. Different analytic formulations are taken depending on the particular characteristics of the vertical profiles for each experimental campaign (see more details in Stiperski *et al*., 2019). The height where the Richardson number increases beyond 0.25 is then chosen as the Stable Boundary Layer (SBL) height ($h_{{\mathrm{Ri}}_{g}}$). This level, although obtained through a qualitative threshold for Ri_g_, compares well with the size of the most energetic eddy determined by multiresolution flux decomposition and measurements show that above this height turbulence is suppressed (Stiperski *et al*., 2020). For the given examples in Figure 2, $h_{{\mathrm{Ri}}_{g}}$ indicates well the height of the maximum jet for the MATERHORN profile and, for METCRAX II, it coincides with the level where the turbulence regime changes.

**Figure 3:**
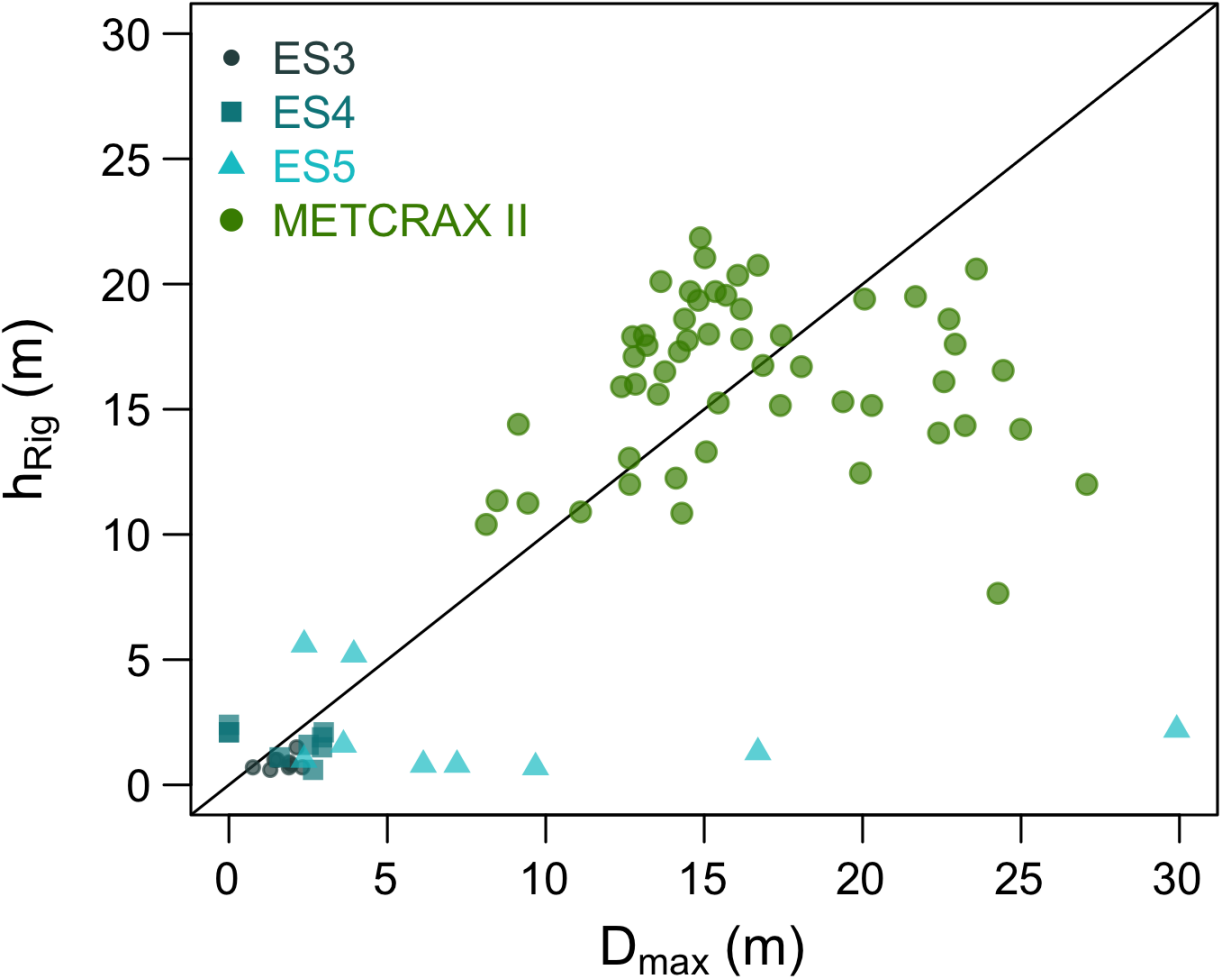
Comparison between SBL height ($h_{{\mathrm{Ri}}_{g}}$), calculated as the first level where the gradient Richardson number increases beyond 0.25 (Ri_g_ > 0.25), and *D*_max_ for selected cases of MATERHORN (towers ES3, ES4, and ES5) and METCRAX II (tower NEAR) datasets

Figure 3 shows the comparison of $h_{{\mathrm{Ri}}_{g}}$ against *D*_max_ for all the half‐hourly averaging periods selected from MATERHORN and METCRAX II. For the latter case, the pure katabatic wind events are chosen by considering those half‐hourly averaging periods of the six IOP nights where *∂θ*_0_/*∂z* > 0.005 K·m^−1^, maximum wind speed is above 5 m·s^−1^ and the surface kinematic heat flux is *H* < −0.005 K·m·s^−1^. When this filter is not applied, the data scattering becomes considerably larger (not shown).

Figure 3 reflects the different length scales involved in the observed katabatic flows, where the larger ones correspond to the METCRAX II cases. There is a good correspondence between the detected SBL height and *D*_max_, although with still some dispersion in the METCRAX II dataset. The points that have values of $h_{{\mathrm{Ri}}_{g}}$ larger than *D*_max_ may be related to entrainment at the top of the downslope flow, a mechanism not considered in the derivation of *D*_max_. MATERHORN towers ES3 and ES4 show a good agreement between length scales while at ES5, the tower over the steepest slope, *D*_max_ is higher in some of the nights. At this tower, the downslope flow may be at a premature stage and it is also more difficult to characterize the stability of the background air.

## 5 CONCLUSIONS

A simple conceptual model for downslope flows is proposed based on the ratio between the cooling rate of the air near the surface to the adiabatic warming that the air experiences flowing downhill. A relation between the depth of the flowing mass, the intensity of the surface cooling represented by the heat flux, the speed of the flow, the slope angle and the background stratification has been derived. If these quantities are at hand, for instance in a numerical model, the depth of the downslope flow may be estimated and used for adequate purposes.

The main driving mechanisms for a downslope flow are well identified in the literature and Expression (7) can be derived analytically within the framework of the layer‐averaged models. However, the thermodynamic equation is often used as a means to reach a solution for the katabatic wind speed and few previous studies focus on the information that can be extracted directly from a simplified form of the heat budget equation for along‐slope flows, as in the present work.

The expression can only be applied for situations when the two main mechanisms (surface cooling and adiabatic warming) prevail and is not to be used in other topographical configuration where mesoscale pressure gradients are relevant, as for valley or basin flows. When computed for well‐defined experimental katabatic flows, as in the MATERHORN and METCRAX II campaigns, the depth provided by the diagnostic model compares generally well with the top of the turbulent stable boundary layer determined through the gradient Richardson number (Ri_g_), consistently with the definition of *D*. The main discrepancies between both height determinations may be related to a transient period or to significant entrainment at the top of the downslope flow, neither of which have been taken into account in the derivation of *D*_max_.
